# Role of Honey in Obesity Management: A Systematic Review

**DOI:** 10.3389/fnut.2022.924097

**Published:** 2022-06-24

**Authors:** Azizah Ugusman, Syarifah Amirah Syed Shahrin, Nurul Hana Azizan, Siva Balan Pillai, Khamini Krishnan, Norizam Salamt, Amilia Aminuddin, Adila A. Hamid, Jaya Kumar, Mohd Helmy Mokhtar

**Affiliations:** Department of Physiology, Faculty of Medicine, Universiti Kebangsaan Malaysia, Kuala Lumpur, Malaysia

**Keywords:** adipose tissue, body mass index, body weight, honey, obesity

## Abstract

Obesity is a metabolic disorder that has become critically prevalent throughout the world. Obesity has been linked to other chronic diseases such as diabetes mellitus, cardiovascular diseases and cancer. Natural products such as honey have been investigated for their potential effect on obesity. Hence, this study systematically reviewed the recent literature concerning the effects of honey on obesity in obese animal models and in people with obesity. The Ovid MEDLINE, PubMed, Scopus, Web of Science and Google Scholar electronic databases were searched for relevant articles. A total of 130 relevant articles were obtained from the initial search. Following a thorough screening, nine articles were selected for data extraction, including six animal studies and three clinical trials. In most of the animal studies, honey demonstrated an anti-obesity effect by reducing body weight, body fat composition and adipocyte size, among others. However, supplementation of honey in clinical trials showed conflicting results. Even though honey supplementation did not demonstrate any weight-reducing effect in some of the clinical trials, none of the trials showed that honey increases body weight. However, the results should be interpreted with caution as most of the studies involved animal models and there is a limited number of high quality, randomized, controlled clinical trials.

**Systematic Review Registration:**

https://inplasy.com/inplasy-2022-6-0038/ PROSPERO, identifier 10.37766/inplasy2022.6.0038.

## Introduction

Obesity and being overweight are pathological conditions characterized by excessive body mass index (BMI) and/or fat mass (1). Overweight is defined as a BMI greater than or equal to 25 kg/m^2^, whereas obesity is defined as a BMI greater than or equal to 30 kg/m^2^ (2). Currently, obesity has reached epidemic proportions worldwide, and the prevalence of obesity is expected to worsen in both developed and developing countries following the changes in dietary patterns and physical activity (3). According to the World Health Organization global status report on non-communicable diseases 2014, 39% of adults are overweight, whereas 13% of people are obese. The percentage of people who are overweight or obese is expected to further increase by 2025 (4).

Obesity is a multifactorial condition with a complicated interplay of genetics and environmental components (1). Convenience and “junk” foods are widely available nowadays and these foods are often highly processed, lack in nutrients, and high in sugar and fat content, all of which can contribute to obesity (5). Factors that contribute to obesity are not only limited to increased calorie intake or decreased physical exercise, but also related to psychological distress and body weight dissatisfaction (6). Additionally, economic progress, increased modernity, urbanization, and globalization have all contributed to the current era's rise in obesity (1).

Obesity results in many pathophysiological complications such as dyslipidemia, hypertension, cardiovascular diseases, diabetes mellitus, and metabolic syndrome. It has also been associated with the development of certain cancers (7). Oxidative stress (8), inflammation (9), and hypoxia (10) are the underlying mechanisms that contribute to obesity-related complications. The main approach to manage obesity is through dietary modification and exercise. Additionally, obesity is managed using pharmacotherapy such as orlistat and bariatric surgery (11). However, both pharmacotherapy and surgical intervention carry certain risks and adverse effects to the patients. Hence, more studies are being conducted to investigate the role of natural products such as honey for managing obesity with minimal side effects (12).

Honey is a natural sweetener that has been regularly used to improve the taste and wholesomeness of food (13). It is a naturally occurring mixture of simple and complex sugars, vitamins, minerals, acids, and enzymes (14). There are about 300 different types of floral honey available globally. The floral source is used to classify different varieties of honey. Among the most known popular varieties of honey include acacia honey, rapeseed honey, buckwheat honey, citrus honey, and multiflora honey (15). Other than that, honey can be classified as raw or processed. Raw honey is more natural and purer than processed honey. Processed honey is heated and bottled in a factory, causing it to lose vitamins, minerals, and other key ingredients (16).

Honey's color, flavor, mineral, and vitamin content are influenced by the flower from which the bees collect the nectar (17). Environmental factors such as flower origin and age, pollen type, climatic factors, and production parameters could influence the quality of honey. Processing methods and storage conditions such as temperature and humidity also impact honey quality (18). Phenolic chemicals are the most common phytochemical substances found in honey (19). Other phytochemical components found in honey include flavonoids, ascorbic acid, catalase, peroxidase, and carotenoids. These components are primarily responsible for honey's antioxidative effect (20). Honey's phytochemical content is also affected by its floral and geographical origins, as well as its processing, handling, and storage (21). The characteristics and phytochemical content of honey are most affected by plant pollen and weather conditions (19).

Honey provides various nutritional benefits such as anti-inflammatory (22), anti-hypertensive, and cardioprotective properties (23, 24), as well as antioxidative effects (25–27). It also possesses antibacterial (28), antifungal (29), antiviral (30), and antitumor (31) activities. Honey has also demonstrated the anti-obesity effects in various studies (1, 32–35). It is postulated that the active components in honey, such as phenol and flavonoid influence fat metabolism by enhancing lipolysis and preventing lipogenesis (36). Hence, this study was conducted to systematically review the relevant studies on the effect of honey on obesity in obese animal models and in people with obesity. This review may provide scientific backup for honey consumption to control body weight.

## Methods

### Search Strategy

The relevant studies were obtained from five online databases, namely, PubMed, Scopus, Ovid MEDLINE, Web of Science and Google Scholar from 1980 until May 2022. The last search was performed on 20th May 2022. The following keywords were used: Honey AND obesity OR overweight OR body weight OR BW OR body mass index OR BMI OR fat mass OR FM OR lean mass OR LM OR waist circumference OR WC OR leptin OR adiponectin OR waist-hip ratio OR WHR. A total of nine relevant articles were finally selected, which include studies by Gohar et al. (1), Rafie et al. (32), Ramli et al. (33), Romero-Silva et al. (34), Samat et al. (35), Mushtaq et al. (36), Farakla et al. (37), Yaghoobi et al. (38) and Erejuwa et al. (39).

### Study Inclusion and Exclusion Criteria

Only full-length original research articles published in English were included. Any clinical (randomized controlled trial) and preclinical (*in vivo*) studies reporting the effect of any type of honey on body weight control in obese animal models or people with overweight or obesity, regardless of the route of administration, dose, and duration of intervention were included. Preclinical studies using non-obese animal models were excluded. As for clinical trials, studies involving subjects with other comorbidities and subjects on pharmacological therapies were excluded. Studies using combined preparation of honey with other therapy such as herbal medicine, review articles, news, case reports, book chapters, conference proceedings, abstract, and editorial letters were also excluded from this review.

### Study Selection and Article Screening

The literature search and articles screening were performed according to the population, interventions, compare, outcome and study design (PICOS) framework, as follows:

- Population (P): Subjects with obesity or overweight as well as animal models of obesity, regardless of animal species, were included.
- Intervention (I): Honey as an intervention in the experimental group were included.
- Comparison (C): The comparator groups received either no intervention or were treated with relevant conventional drug.
- Outcome (O): Changes in body weight, BMI, waist circumference, waist-hip ratio, body fat mass and percentage.
- Study design (S): Clinical (randomized controlled trial) and preclinical (*in vivo*) studies.

The articles retrieved from the databases were independently reviewed by six authors (S.B.P, K.K, S.A.S.S, N.H.A, M.H.M and A.U). Any disagreement was resolved by discussion to reach a consensus. The screening of articles was done in three stages. Firstly, articles that did not meet the selection criteria were rejected only based on their titles. Secondly, studies that were irrelevant to honey and obesity were eliminated by reading the abstracts. Finally, articles that did not meet the inclusion criteria were eliminated after a thorough reading of the full text.

## Results

### Studies Selected

**Figure 1:**
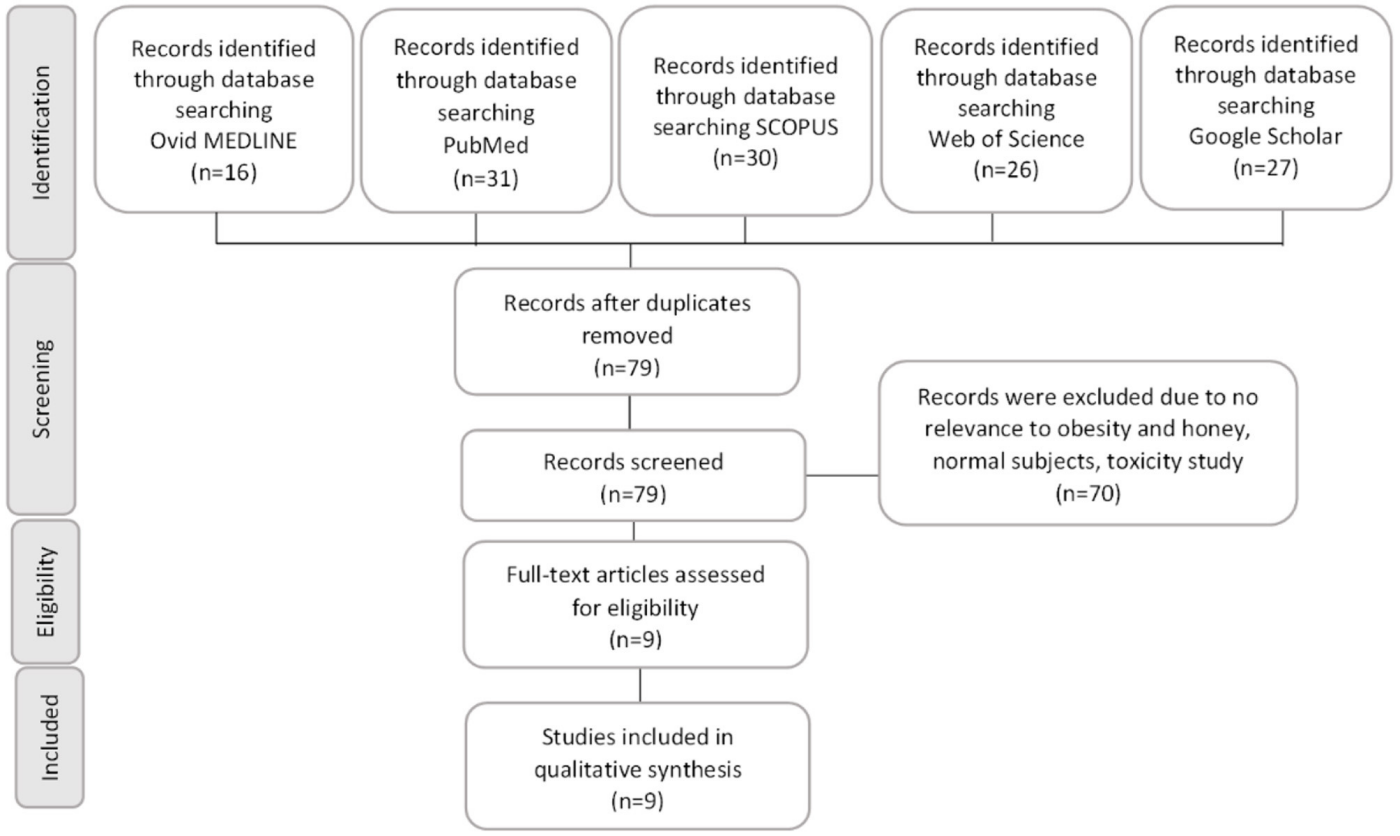
A summary of the literature search and the steps involved in the study selection according to the Preferred Reporting Items for Systematic Reviews and Meta-Analyses (PRISMA) guideline. Overall, nine studies met the search criteria.

A total of 130 articles were found in five online databases, including 16 articles from Ovid MEDLINE, 31 articles from PubMed, 30 articles from Scopus, 26 articles from Web of Science and 27 articles from Google Scholar. Subsequently, 51 articles were removed due to duplication. After reviewing the titles and abstracts, 70 papers were excluded. The remaining nine full-length original articles were collected and thoroughly reviewed. In total, nine articles published between 2008 and 2020 met the search criteria and were included in this review. Figure 1 showed the summary of article selection process. The experimental model, honey type and source, active compound, dose and duration of treatment, findings, and conclusion of each study were listed in Table 1.

**Table 1:** Characteristics of the included studies. Altogether, nine articles were used for data extraction, including six animal studies and three clinical trials.

**Reference**	**Experimental model**	**Honey type(s) / Source(s)**	**Active compound(s)**	**Dose & duration of treatment**	**Findings**	**Conclusions**
(34)	36 female Wistar rats, divided into three groups: -Normal diet -Hypercaloric diet (HCD, given 30% sucrose) -HCD + honey (given 10% sucrose + 20 g butter + honey)	Natural unprocessed honey (unknown type and source)		20 g honey mixed with diet 8 weeks	- Honey did not cause additional weight gain in rats on the HCD regimen compared with the HCD group that received 30% sucrose. - Compared with the rats on HCD with 30% sucrose, rats fed with honey had significantly: Smaller adipocyte size in intra-abdominal fat (62.8 ± 1.6 μm vs. 84.4 ± 2.0 μm, *P* < 0.05) Smaller adipocyte size in mesenteric fat (45 ± 14.5 μm vs. 83.5 ± 1.0 μm, *P* < 0.05)	Honey protects against increased adipocyte size due to a hypercaloric diet.
(35)	30 males, 7-week-old Sprague-Dawley rats, divided into 5 groups: -Normal control -High-fat diet (HFD) -HFD + Gelam honey -HFD + acacia honey -HFD + orlistat	- Gelam honey from Gelam forest, Terengganu, Malaysia - Acacia honey from a farm in Johor, Malaysia	Phenol, Flavonoid	Honey mixed with diet (dose not stated) 4 weeks	- Compared with the HFD group, obese rats fed with Gelam honey had significantly: Lower weight gain (38.39 ± 2.55% vs. 44.34 ± 2.15%, *P* < 0.05) Lower adiposity index (0.85 ± 0.03% vs. 1.13 ± 0.04%, *P* < 0.05) Higher total food intake (1.46 ± 0.07 kg vs. 1.27 ± 0.11 kg, *P* < 0.05) Higher energy efficiency (0.26 ± 0.02 vs. 0.22 ± 0.02, *P* < 0.05) Lower relative weights of liver (2.75 ± 0.14 g vs. 3.26 ± 0.54 g, *P* < 0.05) and lung (0.35 ± 0.02 g vs. 0.39 ± 0.02 g, *P* < 0.05) Lower serum leptin (13.66 ± 0.95 ng/mL vs. 21.70 ± 1.52 ng/mL, *P* < 0.05) and resistin (41.87 ± 1.13 ng/mL vs. 48.39 ± 1.7 ng/mL, *P* < 0.05) Higher adiponectin levels (34.94 ± 1.12 ng/mL vs. 30.51 ± 0.78 ng/mL, *P* < 0.05) - Compared with the HFD group, obese rats fed with acacia honey had significantly: Lower weight gain (40.06 ± 2.40% vs. 44.34 ± 2.15%, *P* < 0.05) Lower adiposity index (0.96 ± 0.02% vs. 1.13 ± 0.04%, P <0.05) Higher energy efficiency (0.24 ± 0.02 vs. 0.22 ± 0.02, *P* < 0.05) Lower relative weights of liver (2.56 ± 0.22 g vs. 3.26 ± 0.54 g, *P* < 0.05), heart (0.29 ± 0.03 g vs. 0.33 ± 0.04 g, *P* < 0.05) and lung (0.34 ± 0.04 g vs. 0.39 ± 0.02 g, *P* < 0.05) Lower serum leptin (12.13 ± 1.23 ng/mL vs. 21.70 ± 1.52 ng/mL, *P* < 0.05) and resistin levels (42.18 ± 1.28 ng/mL vs. 48.39 ± 1.7 ng/mL, *P* < 0.05) - For BMI, both Gelam honey (0.74 ± 0.04 g/cm^2^) and acacia honey (0.75 ± 0.06 g/cm^2^) did not reduce the rats' BMI compared to HFD group (0.77 ± 0.03 g/cm^2^). -Both acacia and Gelam honey showed better effects than orlistat.	Honey can be used to control obesity and is more effective than orlistat.
(32)	48 male, 3-month-old Sprague-Dawley rats, divided into 6 groups: -Normal diet -HFD -HFD + 1000 mg/kg SBH -HFD + 750 mg/kg SBH -HFD + 500 mg/kg SBH -HFD + orlistat	Multiflora stingless bee honey (SBH) from a farm in Kelantan, Malaysia		500, 750, 1,000 mg/kg/day SBH 12 weeks	- Compared with the HFD group, obese rats treated with SBH had significantly: Lower body weight (*P* < 0.05) Lower percentage of body weight gain (P <0.05) Lower BMI (*P* < 0.05) Lower adiposity index (*P* < 0.05) Lower relative liver weight (*P* < 0.05) Lower number of adipocytes in visceral fat on histological examination - SBH demonstrated more desirable effects than orlistat. - SBH dose of 750 mg/kg is the most effective dose to reduce body weight.	SBH could be used as an alternative treatment to combat obesity.
(33)	18 males, 3-months-old Wistar rats, divided into 3 groups: - Normal diet -High- carbohydrate and high-fat diet (HCHFD) -HCHFD + SBH	SBH from a farm in Selangor, Malaysia	4-hydroxyphenyl acetic acid, caffeic acid derivative, caffeoyl hexoside derivative, coumaric acid, gluconic acid, kynurenic acid derivative, pinobanksin, quinic acid, niazimicin, bisosthenon B, (6β,7α,12β,13β)-7-hydroxy-11,16-dioxo-8,14-Apianadien-22,6-olide, aegle marmelos alkaloid C, 7-chloro-6-demethylcepharadione B, n-acetylglycine and lanosterol	1 g/kg/day SBH via oral gavage 8 weeks	Compared with the HCHFD group, obese rats fed with SBH had significantly: Lower body fat percentage (18.40 ± 2.15% vs. 23.48 ± 2.10%, *P* < 0.05) Lower omental fat mass (40.06 ± 2.40% vs. 44.34 ± 2.15%, *P* < 0.05) Lower adipocyte area (1920.97 ± 45.49 μm^2^ vs. 3931.73 ± 348.79 μm^2^, *P* < 0.05) Lower adipocyte parameter (176.45 ± 2.13 μm vs. 234.75 ± 11.62 μm, *P* < 0.05) - No significant reduction in body weight in the group fed with SBH compared to HCHFD rats (318.10 ± 17.58 g vs. 352.42 ± 14.57 g, *P* > 0.05).	SBH reverses HCHFD-induced increase in body fat.
(1)	36 male Wistar rats, divided into 6 groups: -Normal diet + saline -Normal diet + 1 g/mL/kg honey -Normal diet + 2 g/mL/kg honey -HFD + saline -HFD + 1 g/mL/kg honey -HFD + 2 g/mL/kg honey	Acacia Honey from local beekeepers in Karachi, Pakistan		1, 2 g/mL/kg honey orally 4 weeks	- Compared with the saline-treated HFD group, obese rats treated with high and low doses of acacia honey had significantly: Lower body weight in dose-response fashion (*P* < 0.01) Higher locomotor activity (*P* < 0.01) - No significant changes in caloric intake of groups treated with high and low doses of acacia honey compared to saline-treated group	Acacia honey reverses the adverse effects of HFD on body weight gain and locomotor activity.
(39)	25 male Wistar rats, divided into 5 groups: -Normal chow diet + 1 mL/kg BW drinking water -HFD + 30% sucrose + 1 mL/kg BW drinking water -HFD + 30% sucrose + 1 g/kg BW honey -HFD + 30% sucrose + 2 g/kg BW honey	Honey from a bee farm in Ebonyi State, Nigeria		1, 2, 3 g/mL/kg honey orally 6 weeks	-Compared with the untreated HFD group, obese rats fed with 1 g/kg BW honey had significantly: Lower BMI (*P* < 0.01) Lower % change in BMI (*P* < 0.01) Lower body weight/body length ratio (*P* < 0.05) Lower adiposity index (*P* < 0.05) Lower % change in adiposity index (*P* < 0.05) -Honey-treated obese rats showed no significant difference in BMI, body weight/body length ratio and adiposity index compared to rats fed with normal diet.	Honey produces beneficial effects on obesity anthropometric parameters.
	-HFD + 30% sucrose + 3 g/kg BW honey -Normal diet + 1 g/mL/kg honey -Normal diet + 2 g/mL/kg honey -HFD + saline -HFD + 1 g/mL/kg honey -HFD + 2 g/mL/kg honey					
(37)	30 obese prepubertal girls (aged 10 ± 0.34 years, BMI above the 97th centile for age; 28.58 ± 1.40 kg/m^2^, BMI z-score 2.96), divided into 2 groups: -Control (N = 15, given marmalade)	Wildflowers-forest thyme honey, Greece	Phenol	15 g honey daily, orally 6 months	-Subjects in both control and honey-supplemented groups had a significant reduction in their body weight and BMI after 6 months. -Compared to the control group, the reduction in body weight and BMI in the honey-supplemented group was not significant (body weight reduction 0.62 ± 1.13 kg vs. 1.42 ± 0.72 kg, *P* = 0.467; BMI reduction−1.06 ± 0.38 vs.−0.96 ± 0.28, *P* = 0.715).	Honey does not influence the body weight in obese prepubertal girls.
	-Experiment (N = 15, given honey) Both groups were on dietary restriction and a regular exercise regime.					
(36)	80 obese adults (40 males and 40 females from four different ethnicities, BMI ≥ 30 kg/m^2^) divided into 2 groups: -Control (*N* = 40) -Experiment (N=40, given honey) The subjects maintained their regular dietary and physical activity habits.	Alshifa Natural Honey from Jeddah, Saudi Arabia		40 g daily, orally 4 weeks	-Honey supplementation did not cause significant BMI reduction compared to the control group in all ethnicities in both genders.	Natural honey does not reduce BMI in obese adults.
(38)	60 overweight or obese (BMI > 25 kg/m^2^) adults, divided into 2 groups: -Control (N = 17, given sucrose) -Experiment (N = 38, given honey) Subjects in both groups did not undergo any special diet, drug therapy or exercise regime throughout the study.	Natural unprocessed honey (unknown type and source)		70 g daily, orally 30 days	-Compared to the control group, honey-supplemented group had significantly lower BMI (29.8 ± 3.2 kg/m^2^ vs. 32.8 ± 5.0 kg/m^2^, *P* = 0.02). -Honey supplementation did not cause significant change in body weight, body fat weight and percentage, and waist circumference compared to the control group.	Natural honey does not increase the body weight in overweight and obese subjects.

*BMI, Body mass index; EE, Energy expenditure; HCD, Hypercaloric diet; HCHFD, High-carbohydrate and high-fat diet; HF, Honey-fed; HFD, High-fat diet; ND, Normal diet; SBH, Stingless bee honey*.

### Experimental Models

The studies selected consisted of three clinical trials and six animal studies. The experimental models used in the animal studies were diet-induced obese Wistar rats (1, 33, 34, 39) and Sprague Dawley rats (32, 35). The control rats were fed a normal chow diet, while obesity in rats were induced with a hypercaloric diet (HCD) (34), high-fat diet (HFD) (1, 32, 35, 39), and high-carbohydrate and high-fat diet (HCHFD) (33). Some of the animal studies used orlistat as a positive control (32, 35). For clinical trials, the subjects involved were obese prepubertal girls (37) and obese adults (36, 38). Control groups in the clinical trials were supplemented with marmalade (37) and sucrose (38). The duration of honey supplementation varied among the clinical and animal studies. The animal studies involved a shorter duration of honey supplementation between four and 12 weeks, compared to clinical trials, which had a duration of 4 weeks to 6 months. For the animal studies, the doses of honey used varied between 20 and 3,000 mg/kg/day, while the doses used in clinical trials ranged between 15 and 70 g daily.

### Types, Sources and Contents of Honey

Malaysia produces various honey, including flower honey (such as honey harvested from trees like Gelam and Tualang trees) and honeydew honey (such as acacia honey), owing to its tropical climate and wealth of floral sources (40). Three of the animal studies used three different types of honey from Malaysia (32, 33, 35). One of them used Gelam honey from Gelam Forest, Terengganu and acacia honey from a farm in Johor (35), while two other studies used stingless bee honey (SBH) obtained from two different states in Malaysia, namely, Kelantan (32) and Selangor (33). Four other animal studies used wild flowers-forest thyme honey from Greece (37), acacia honey from Karachi, Pakistan (1), Alshifa natural honey from Jeddah, Saudi Arabia (36) and honey from Ebonyi State, Nigeria (39). Meanwhile, in the remaining two animal studies, the type and source of the natural honey used were not stated in the articles (34, 38). The active compounds found in honey are phenols (35, 37) and flavonoids (35). One study conducted gas chromatography-mass spectrometry (GC-MS) analysis of SBH and found various active compounds in SBH such as 4-hydroxyphenyl acetic acid, caffeic acid derivatives, caffeoyl hexoside derivatives, coumaric acid, gluconic acid, kynurenic acid derivatives, pinobanksin, quinic acid, niazimicin, bisosthenon B, (6β,7α,12β,13β)-7-hydroxy-11,16-dioxo-8,14-apianadien-22, 6-olide, aegle marmelos alkaloid C, 7-chloro-6-demethyl cepharadione B, n-acetylglycine and lanosterol (33).

### Effects of Honey on Obesity

Most of the animal studies have demonstrated that honey has weight-reducing properties, as evidenced by lower body weight, percentage of body weight gain, and BMI (1, 32, 35, 39). However, in two of the animal studies, honey supplementation did not cause a significant reduction or increment in body weight in the obese rats (33, 34). In terms of body fat, honey supplementation lowered the body fat percentage (33), adiposity index (32, 33, 35, 39), and omental fat mass (33). Microscopically, honey consumption led to a lower adipocyte area and adipocyte parameter (33), reduced number of adipocytes in the visceral fat (32) and smaller adipocyte size in the intra-abdominal and mesenteric fats (32, 34).

Honey consumption also decreased the internal organ weight such as the heart (35), lung (35), and liver (32). In terms of adipokine profile, serum adiponectin increased, while serum leptin and resistin decreased when obese rats were fed with honey (35). Furthermore, obese rats that consumed honey also had an increase in locomotor activity (1), and a higher energy efficiency (35), even though their caloric intake remained the same (1). Gelam honey, acacia honey and SBH were more effective in controlling obesity compared to orlistat (32, 35).

Meanwhile, clinical trials have shown conflicting results with the animal studies (36–38). Men and women with overweight and obesity who received honey supplementation had significantly lower BMI compared to the control group who received sucrose. However, there were no significant changes in their body weight, body fat weight and percentage, and waist circumference compared to the control group (38). In another trial involving obese prepubertal girls (37), both control and honey-supplemented groups had significant reductions in their body weight and BMI after six months. Both control and honey-supplemented groups were on strict dietary control and exercise. Nevertheless, the reduction in body weight and BMI in the honey-supplemented group was not significant compared to that in the control group. Additionally, honey did not have any effect on postprandial glucose and insulin levels (37). Honey supplementation also did not cause significant BMI reduction in obese men and women from the four different ethnicities (36). Even though honey supplementation did not cause a significant reduction in the subjects' body weight, there was no evidence showing that honey increased their body weight (36–38).

## Discussion

This study systematically reviewed current studies related to the effect of honey from various sources on obesity in animal studies and clinical trials involving obese subjects. Most of the obesity induction in animal models was achieved through the consumption of HFD as HFD mimicked the common cause of obesity in humans (41). HFD generates a positive energy balance as it is not only high in calories, but it also reduces energy expenditure (1). A positive energy balance results in increased visceral fat deposition and subsequently, abdominal obesity (42). Wistar rats were used in some studies because they acquired weight more quickly when fed with HFD and represented a polygenic diet-induced obesity model (43). Two studies used 7-week-old (35) and three-month old Sprague Dawley rats (32), respectively, which are the favorable age for studying obesity in animal model (44, 45).

Honey demonstrated various anti-obesity actions in animal studies by reducing body weight, BMI, body fat composition, fat mass, adipocyte area, and adipocyte size. The weight-reducing effect of honey could be explained by several mechanisms. Mono-and disaccharides such as glucose, fructose, maltose, and sucrose make up most of honey's composition. These simple sugars are quickly absorbed and metabolized. About 35–45% of honey is made up of fructose (46–48), which delays gastric emptying (49) and therefore, reduces food consumption (50). Honey also produces a laxative effect *in vivo* (47). The laxative effect leads to the rapid removal of water and waste from the body (51). This effect directly reduces body water content but not fat (51), which indirectly reduces body weight because water makes up majority of body composition. It has also been suggested that honey reduces body weight by promoting lipolysis and preventing lipogenesis (36). Besides, glucose oxidase enzyme catalyzes the formation of hydrogen peroxide from glucose in honey (35). Hydrogen peroxide has insulin-mimetic action that improves the metabolic rate, hence contributing to weight loss with honey consumption (5).

The body's energy homeostasis is shifted to a positive energy balance when energy intake exceeds energy expenditure, which promotes adipose tissue gain and leads to obesity (6, 52, 53). A larger calorie intake should theoretically lead to more weight gain, but this is not the case with honey consumption. Supplementation with Gelam honey in HFD-induced obese rats resulted in a higher total food intake, higher energy efficiency, and lower weight gain (35). The higher energy efficiency with honey intake causes the excess food to be turned into energy rather than for body fat storage, hence causing honey-fed rats to gain less weight despite consuming more food (35). A previous study also showed that locomotor activity positively correlates with energy expenditure and is an important contributor to body weight control (54). Decreased locomotor activity leads to a low energy expenditure in HFD-induced obese rats (1). Interestingly, honey supplementation led to a higher locomotor activity in obese rats that caused a greater energy expenditure and consequently weight loss (1).

Honey also influences body weight by modifying adiposity levels. Adipocyte hypertrophy (increased adipocyte size), adipocyte hyperplasia (increased adipocyte number), or a combination of the two factors may cause the expansion of adipose tissue in obesity (55, 56). Adipocyte hypertrophy is the major contributor of increased fat mass in obesity compared with adipocyte hyperplasia. Adipocyte hyperplasia does not contribute much to fat mass because the newly created cells have limited area for fat storage (57). Honey was shown to reduce adipocyte hypertrophy in HCD-induced obese rats (34). In other studies, SBH reduced the area covered by adipocytes in HCHFD-induced obese rats (35), and decreased adipocyte hyperplasia in the visceral fat of HFD-induced rats (32). However, the exact mechanism by which honey lowers adipocyte hypertrophy and hyperplasia remains unclear (32).

The number and size of adipocytes also affect the adiposity index and relative organ weight that eventually affect the body weight. The adiposity index is a method used to measure the body fat distribution (58). It was calculated by dividing the total weight of epididymal, visceral and retroperitoneal fat with the body weight (32). Adipose tissue is an effective buffer against daily lipid fixation in the systemic circulation. When the buffering ability of adipose tissue is compromised, tissues such as skeletal muscles and liver tissue are exposed to lipid accumulation (59). Acacia honey and Gelam honey reduced the relative weight of the liver, heart, and lungs of HFD-fed rats (32). Furthermore, Gelam honey, acacia honey and SBH reduced the adiposity index of obese rats (32, 35). All these factors contribute to the weight-reducing effect of honey.

Adipokines such as adiponectin, leptin, and resistin are released by adipose tissue and their production is altered in obesity. Leptin is a fat-derived key regulator of appetite and energy expenditure, and its plasma concentration is linked to adiposity (60). Resistin is a pro-inflammatory adipokine that suppresses glucose uptake and insulin sensitivity (61). On the other hand, adiponectin is an anti-inflammatory adipokine that enhances insulin sensitivity (62). Obese rats showed increased levels of resistin and leptin, and reduced levels of adiponectin. Supplementation of obese rats with Gelam honey and acacia honey successfully reduced the levels of leptin and resistin, while the adiponectin level was increased (35). These findings suggest that honey causes a significant adipocyte loss, as evidenced by lower leptin and resistin levels, whereas the increased adiponectin levels reduce the weight gain in rats fed with honey (35).

Previous studies have demonstrated that central obesity triggers low-grade systemic inflammation, as evidenced by elevated inflammatory markers such as C-reactive protein, tumor necrosis factor-α, and interleukin-6 in the plasma of people with central obesity (63–65). Systemic oxidative stress and chronic inflammation play a critical role in sustaining obesity (66). Gelam honey and acacia honey contain phenolic acids and flavonoids, which are powerful antioxidative and anti-inflammatory compounds (35). Table 2 summarizes the common phenolics acids and flavonoids found in different types of honey. For example, SBH contains coumaric and caffeic acids as its active components. Both coumaric and caffeic acids have anti-obesity effects (81–83).

**Table 2:** Common phenolic acids and flavonoids found in different types of honey.

**Bioactive compound**	**Types of honey**	**Potential health benefits**
Quercetin C_15_H_10_O_7_	Gelam Honey (67) Stingless Bee Honey (68) Acacia Honey (69) Thyme Honey (69)	Anti-allergy, anti-inflammation, antioxidative, anti-proliferation, anti-obesity and anti-tumor (70)
Kaempferol C_15_H_10_O_6_	Gelam Honey (71) Stingless Bee Honey (68) Acacia Honey (27) Thyme Honey (69)	Anti-tumor, antioxidative and anti-inflammation (72)
Genistein C_15_H_10_O_5_	Acacia Honey (69)	Antioxidative, anti-inflammation, anti-bacterial, and anti-viral (73)
Apigenin C_15_H_10_O_5_	Acacia Honey (69) Gelam Honey (71) Stingless Bee Honey (68)	Anti-inflammation, anti-mutagenic, cardioprotective activity (74)
Chrysin C_15_H_10_O_4_	Acacia Honey (69) Thyme Honey (69) Stingless Bee Honey (68) Gelam Honey (67)	Antioxidative, anti-inflammation, anti-apoptosis, anti-cancer, and neuroprotective (75)
Gallic Acid C_7_H_6_O_5_	Gelam Honey (67) Stingless Bee Honey (68) Acacia Honey (69) Thyme Honey (69)	Antioxidative, anti-inflammation, anti-mutagenic, anti-cancer and cardioprotective activity (76)
Caffeic Acid C_9_H_8_O_4_	Gelam Honey (67) Stingless Bee Honey (68) Acacia Honey (69) Thyme Honey (69)	Antioxidative, anti-inflammation, anti-obesity and anti-carcinogenic (77)
Chlorogenic acid C_16_H_18_O_9_	Gelam Honey (67) Acacia Honey (69) Thyme Honey (69)	Anti-diabetes, anti-carcinogenic, anti-inflammation and anti-obesity (78)
p-Coumaric acid C_9_H_8_O_3_	Gelam Honey (67) Stingless Bee Honey (68) Acacia Honey (69) Thyme Honey (69)	Antioxidative, anti-inflammation, anti-obesity, anti-diabetes, anti-ulcer, anti-platelet, anti-cancer (79)
Ferulic acid C_10_H_10_O_4_	Gelam Honey (67) Acacia Honey (69) Thyme Honey (69)	Anti-inflammatory, antioxidant, anti-microbial, anti-cancer, and anti-diabetes (80)

In 3T3-L1 adipocytes, coumaric acid triggers G1 cell cycle arrest (84), whereas caffeic acid inhibits fatty acid synthesis (85). Additionally, gelam honey, SBH, acacia honey and thyme honey contain the flavonoid quercetin, which is a powerful antioxidant (67–69). Antioxidants could scavenge free radicals by inhibiting some enzymes or by chelating trace metals (86). It may provide a viable therapeutic approach to alleviate the excessive reactive oxygen species production, oxidative stress, and adverse effects of obesity. Quercetin reduces oxidative stress associated with obesity by modulating the mitogen-activated protein kinase (MAPK) and adenosine monophosphate-activated protein kinase (AMPK) pathways (87). Quercetin inhibits adipogenesis in 3T3-L1 adipocytes by activating AMPK (88) and attenuates macrophage infiltration and inflammation in the adipose tissue of obese mice by enhancing AMPK and sirtuin-1 expression (89). Clinically, consumption of 150 mg/day of quercetin by overweight and obese men with various apolipoprotein E genotypes successfully decreased their waist circumference (90). In short, the anti-obesity effect of honey is most likely mediated by its phenolic acids and flavonoid contents that possess antioxidative and anti-inflammatory activities.

Even though honey has demonstrated promising anti-obesity effects in animal studies, most clinical trials showed that it has no significant weight-reducing effect. Unlike animal studies, confounding factors that can affect body weight such as diet and physical activities, were not controlled in the clinical trials (36, 38). Besides, the clinical trials only include a small sample size, which are 30 obese prepubertal girls (37), 80 obese adults (36) and 60 overweight and obese adults (38). Additionally, the duration of the clinical trials was short, between 30 days (38) to 4 weeks (36). It has been proven that a well-designed, randomized, controlled clinical trial is the most reliable method to determine the effectiveness of an intervention as it reduces the chance of confounding factors from affecting the results (91). Therefore, to determine the effect of honey on obesity in humans, more high-quality, randomized, controlled clinical trials are needed.

Nevertheless, the prevalence of obesity differs with sex and racial ethnic identity. For instance, in the United States of America, the prevalence of obesity is highest among non-Hispanic Blacks and Hispanics, and lower among Asians and non-Hispanic whites (92). In most countries, obesity is more prevalent in women than men (93). This pattern has been attributed to genetic factor that affect body composition and fat distribution, as well as socioeconomic factors (94, 95). Only one clinical trial evaluated the effect of honey on the BMI of obese men and women from four different ethnic groups in Pakistan: Baloch, Pathan, Hazara and Punjabi. The results showed that honey supplementation did not cause any significant BMI reduction in obese men and women of all four ethnicities (36). However, the participants were not subjected to any dietary control or physical activity regime. Further studies involving different ethnic backgrounds are needed to ascertain the role of different sex and race on the effect of honey in obesity.

## Conclusion

Honey exerts anti-obesity effects in animal studies by reducing body weight, BMI, body fat composition, adiposity index, adipocyte hypertrophy and adipocyte hyperplasia. However, most clinical trials show insignificant results due to the small sample size, limited treatment duration and the presence of confounding factors such as diet and physical activity. Therefore, more high quality, randomized, controlled clinical trials are needed to establish the effect of honey in obese humans.

## Data Availability Statement

The original contributions presented in the study are included in the article/supplementary material, further inquiries can be directed to the corresponding author/s.

## Author Contributions

AU, JK, and MM contributed to conception and design. SS, NA, SP, KK, AA, AH, and NS contributed to data acquisition. SS, NA, SP, KK, NS, and AU were involved in analysis and interpretation and drafting the manuscript. AU, JK, AA, AH, and MM revise the manuscript critically for important intellectual content. All authors contributed to the article and approved the submitted version.

## Funding

This research was funded by Faculty of Medicine, Universiti Kebangsaan Malaysia (grant code FF-2021-235) under the Special Study Module.

## Conflict of Interest

The authors declare that the research was conducted in the absence of any commercial or financial relationships that could be construed as a potential conflict of interest.

## Publisher's Note

All claims expressed in this article are solely those of the authors and do not necessarily represent those of their affiliated organizations, or those of the publisher, the editors and the reviewers. Any product that may be evaluated in this article, or claim that may be made by its manufacturer, is not guaranteed or endorsed by the publisher.
